# Test-retest reliability of knee extensors endurance test with elastic resistance

**DOI:** 10.1371/journal.pone.0203259

**Published:** 2018-08-31

**Authors:** Jaqueline Santos Silva Lopes, Jéssica Kirsch Micheletti, Aryane Flauzino Machado, Larissa Rodrigues Souto, Heloísa Paes de Lima, Franciele Marques Vanderlei, Jayme Netto Junior, Carlos Marcelo Pastre

**Affiliations:** São Paulo State University (Unesp), School of Technology and Sciences (Department of Physical Therapy), Presidente Prudente São Paulo, Brazil; National Center of Medicine and Science in Sport, TUNISIA

## Abstract

**Background:**

Reliable clinical tests capable of measuring resistance are important tools for rehabilitation. One alternative that has recently increased in popularity is the use of elastic tubes, which stand out for being easy to handle, low cost, practical, and feasible.

**Objective:**

Analyze the test-retest reliability of the knee extensors muscle fatigue resistance test (FRT) with elastic tubes.

**Methods:**

A total of 116 healthy young males, aged between 18 and 30 years old, participated in the study. Participants performed three pre-test stages: orientation, load presentation, and familiarization with equipment, lasting two weeks. Subsequently, they performed the FRT on two occasions (test and retest), with an interval of seven days. The reliability analyzes were performed using the intraclass correlation coefficient (ICC) with 95% confidence interval and typical measurement error (TME), also expressed as coefficient of variation (CV%).

**Results:**

The findings regarding the reliability of the test demonstrated satisfactory values (time: ICC = 0.66; 95%CI [0.50; 0.76]; CV(%) = 9.34; repetition: ICC = 0.61; 95%CI [0.46; 0.73], CV(%) = 13.66; rhythm: ICC = 0.52; 95%CI [0.35; 0.67], CV(%) = 10.29.

**Conclusion:**

From the findings presented, it is concluded that the proposed clinical test with elastic tubes demonstrates evidence of acceptable values.

## Introduction

The use of elastic resistance has increased in recent years in training and physical rehabilitation programs. This alternative method has demonstrated similar evidence in different populations and outcomes when compared to traditional tools, such as weight machines and free weights. Compared with other traditional tools, the use of elastic resistance achieves positive and similar results in several outcomes and populations, in addition to which elastic devices (bands and tubes) stand out for being easy to handle, low cost, practical, and feasible to use [1–4].

Despite the widespread use, the load dynamics for exercise with elastic resistance remain questionable considering the prescription. In general, recommendations for performing resistance exercises adopt parameters to implement load, complexity, speed, time, and frequency. However, these recommendations are not being applied in exercises with elastic resistance. Studies consider the exercise prescriptions based on tests [5] of gradual evolution without an initial parameter [6] or without changes in load [7], a scenario which comprises a lack of standardization of protocols that use elastic resistance and characterizes a problem to be solved since the lack of important parameters does not take into account the basic principles of training, such as the biological individuality, overload, and adaptability. Therefore, understanding elastic resistance as an alternative for functional gains together with a definition of parameters seem to be conditions to be respected when prescribing elastic resistance training.

Resistance tests may be an option for assessing the clinical evolution of dysfunctions that have repercussions on body segments, specifically related to fatigue. Strategies using results of resistance tests to prescribe localized exercises may form the beginning of a rehabilitation process prior to the prescription of higher loads. Therefore, reliable tests are important tools in the clinical context, also providing parameters for evolution and control of external training load or rehabilitation programs.

The low exploration of this theme in the scientific literature is evident. Only six studies have investigated the reliability of tests performed using elastic resistance, which all verified maximum strength from maximum repetitions, in populations ranging from healthy individuals to the elderly [8–13]. On the other hand, to our knowledge this is the first study to verify a test of muscle resistance using elastic tubes where the investigated variables, time and repetitions, are specific and characteristics for activities that recruit the physical ability related to muscle endurance.

The described condition comprises a lack in the standardization of protocols that use elastic resistance and characterizes a problem to be solved, since this condition does not respect the principles of training, such as the biological individuality and adaptability. Therefore, the investigation of clinical alternatives for reproducible evaluation methods is pertinent, principally tests that evaluate the resistance condition. Thus, the purpose of this study was to analyze the reliability of the muscle fatigue resistance test (FRT) with elastic tubes in knee extensors in healthy adults.

## Methods

### Participants

The sample consisted of 116 healthy young males. The recruitment of participants was carried out through ads on social networks. During data collection, motivation and daily reminders were sent by electronic messages to minimize the dropout rate. The characteristics of the participants are shown in Table 1.

**Table 1:** Characteristics of the sample (mean ± SD).

	ER (*n* = 81)
**Age (years)**	22.6 ±3.6
**Height (m)**	1.76 ± 0.0
**Weight (kg)**	75.9±16.2
**BMI (kg/m**^**2**^**)**	24.2±4.1

**ER:** Elastic Resistance; **SD:** Standard Deviation; **BMI:** Body Mass Index. p<0.05.

**Fig 1:**
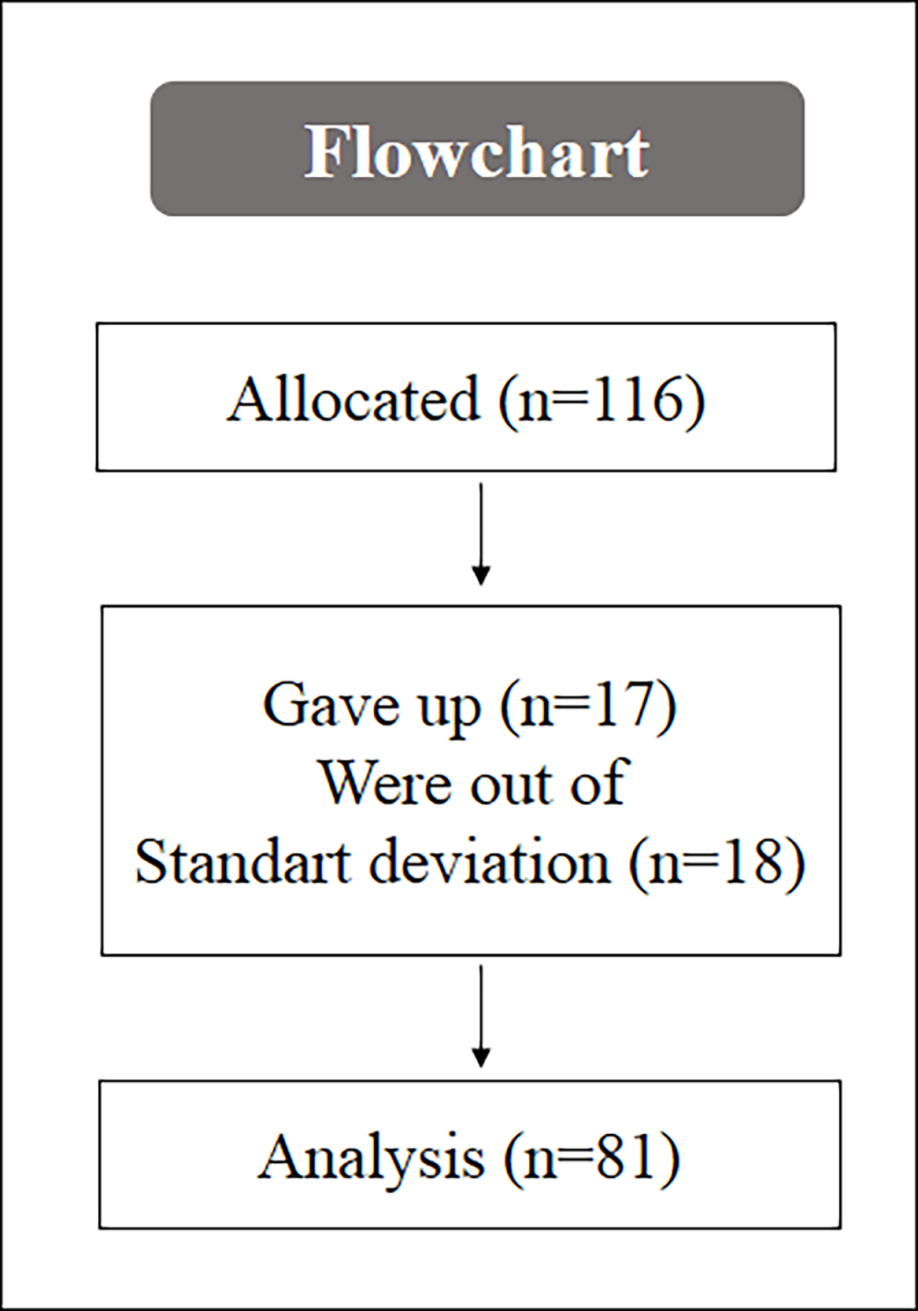
Flowchart of the study. Legend: n = number participantes.

Inclusion criteria were active young people, healthy, aged between 18–30 years, and with full knee flexion-extension amplitude (90°). Participants with the following characteristics were excluded: alcoholism, smoking, chronic use of drugs or anti-inflammatory medication, the presence of anemia, inflammatory process, diabetes, cardiovascular disease, and musculotendinous injury or osteoarticular injury in the lower limbs and/or spine in the previous six months. In addition, participants who dropped out of the study for any reason or cases of injury during daily activities in the collection period and those (a posteriori) who presented values higher than two standard deviations in the variable time were excluded in order to eliminate outliers from the analysis. Fig 1 represents the flowchart of the study.

### Ethics statement and clinical trial registry

Participants were informed about the procedures and objectives of the study and, after agreeing, signed a consent form. All procedures were previously approved by the Ethics Committee in Research of the São Paulo State University (Faculty of Science and Technology—UNESP; protocol number: 2015/322.535) and registered in Clinicaltrials.gov: NCT02764840.

### Study design

The study was conducted by the Center for Studies and Attendance in Physiotherapy and Rehabilitation (CEAFIR), Faculty of Sciences and Technology of the São Paulo State University (UNESP), Presidente Prudente Campus. The study protocols were registered online in the protocols.io, dx.doi.org/10.17504/protocols.io.kckcsuw[PROTOCOL DOI].

Each participant completed six visits during the study, all at the same time of day and in standard conditions. Participants were instructed not to perform vigorous exercise for 24 hours before data collection and to eat a light meal at least two hours before the procedures.

The procedures were performed on the dominant lower limb, with full knee flexion-extension amplitude (90°). Elastic tubes of thickness 11.5 x 6 mm and 9 x 6 mm of the brand Lemgruber (Rio de Janeiro, Brazil) were used.

The study was composed of five stages: 1) Orientation; 2) load presentation; 3) Learning and Standardization; 4) Test; 5) Retest. The interval period between stages 1, 2, and 3 was from 48 to 72 hours and between steps 3, 4, and 5 seven days.

After performing the tests, the perceived exertion scale for resistance exercise (OMNI-RES) [14] was applied.

Before the procedures, the elastic tubes were tested to guarantee homogeneity and reliable results. Any tubes considered different were excluded. The procedure performed for tube verification is described in the section entitled "Selection of elastic tubes". In stages 1, 2, and 3 the same tubes were used for each participant. In stages 4 and 5, new tubes were used.

### Stages of the study

#### Stage 1 –Orientation

Information regarding body mass (Tanita BC 554, Iron Man / Inner—Illinois, USA) and height (Sany—American Medical do Brasil, São Paulo, Brazil) was collected. Next, the participants performed two sets of 20 seconds with an individual load classified as "easy" (2 points) on the OMNI-RES scale [15].

#### Stage 2 –Load presentation

Participants performed the extension movement using the six possibilities available (S1 Chart) in one set of 20 seconds with two intermediate resistances (described in the procedures topic).

#### Stage 3 –Learning and standardization

Stage 3 consisted of two test simulation sessions aiming to familiarize participants and verify the appropriate resistance according to biological individuality. The participants started the procedures with the resistance tube (100 cm, with a 9 mm x 6 mm diameter tube) (S1 Chart). The physical therapists and participants were blinded regarding the resistance used. An independent research assistant stored data and adjusted the appropriate distance to perform the described procedures.

#### Stage 4 –Test

Participants performed the FRT with the pre-defined load from stage 3. The test results, including time and repetition, were not revealed to the participant and an independent research assistant recorded all information.

#### Stage 5 –Retest

Again, in a single session, supervised by the same physical therapists as the previous stage, the participants performed the test with the same load to that used in stage 4. This stage is characterized by analysis of test-retest reliability.

**Fig 2:**
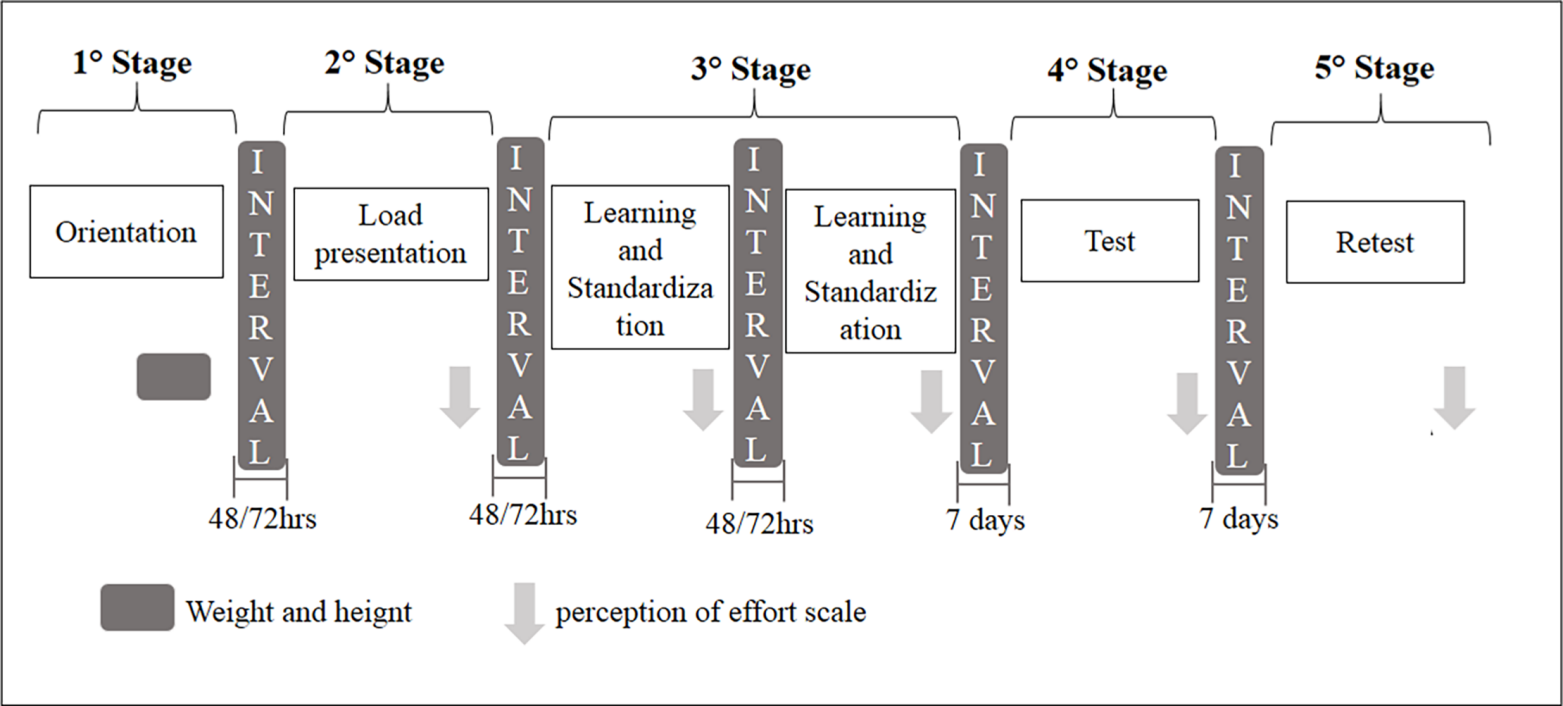
Study design. MIVC: Maximum Isometric Voluntary Contraction; OMNI-RES: perceived exertion scale for resistance exercise.

The study design is shown in Fig 2.

### Procedures

#### Fatigue resistance test (FRT)

The FRT aims to evaluate the local muscular resistance of knee extensors for subsequent training prescription.

The test is based on the energy expenditure theory with the anaerobic glycolytic system. The test was performed for between 40 and 75 seconds, characterized as sufficient for fatigue. It is expected that the test will be performed at high intensity, but with a safe interval and without significant lactate accumulation [15].

If the participant is able to perform the test for a longer time, the proposed resistance is characterized as very light. On the other hand, if fatigue occurs before 40 seconds, the resistance is characterized as beyond functional capacity. When the test is not performed in the estimated time interval, a new attempt is made with different resistance. Ramos et al. [16] used the test protocol described above in a previous study.

The participants were instructed to perform maximum repetitions, free of signs and symptoms, at the highest possible coordinative velocity and maintaining the rhythm from the beginning of the test. The following criteria for interruption of the test were used: reduction in the range of motion, any compensation, decrease in rhythm, and execution time lower than 40 seconds or greater than 75 seconds. The evaluator was responsible for assessing compliance with the requirements described.

Previous pilot studies have identified different levels of resistance required for the population analyzed (Supporting Information).

#### Execution of FRT

Before the test, participants performed the procedures with the elastic tube to learn the movement. One end of the tube was fixed to a hook on an iron bar fixed to the wall in the vertical position, and the other end remained movable, secured with velcro wrapped around the ankle of the participant. The anatomical reference point for velcro placement corresponded to 4 centimeters (cm) above the medial malleolus.

**Fig 3:**
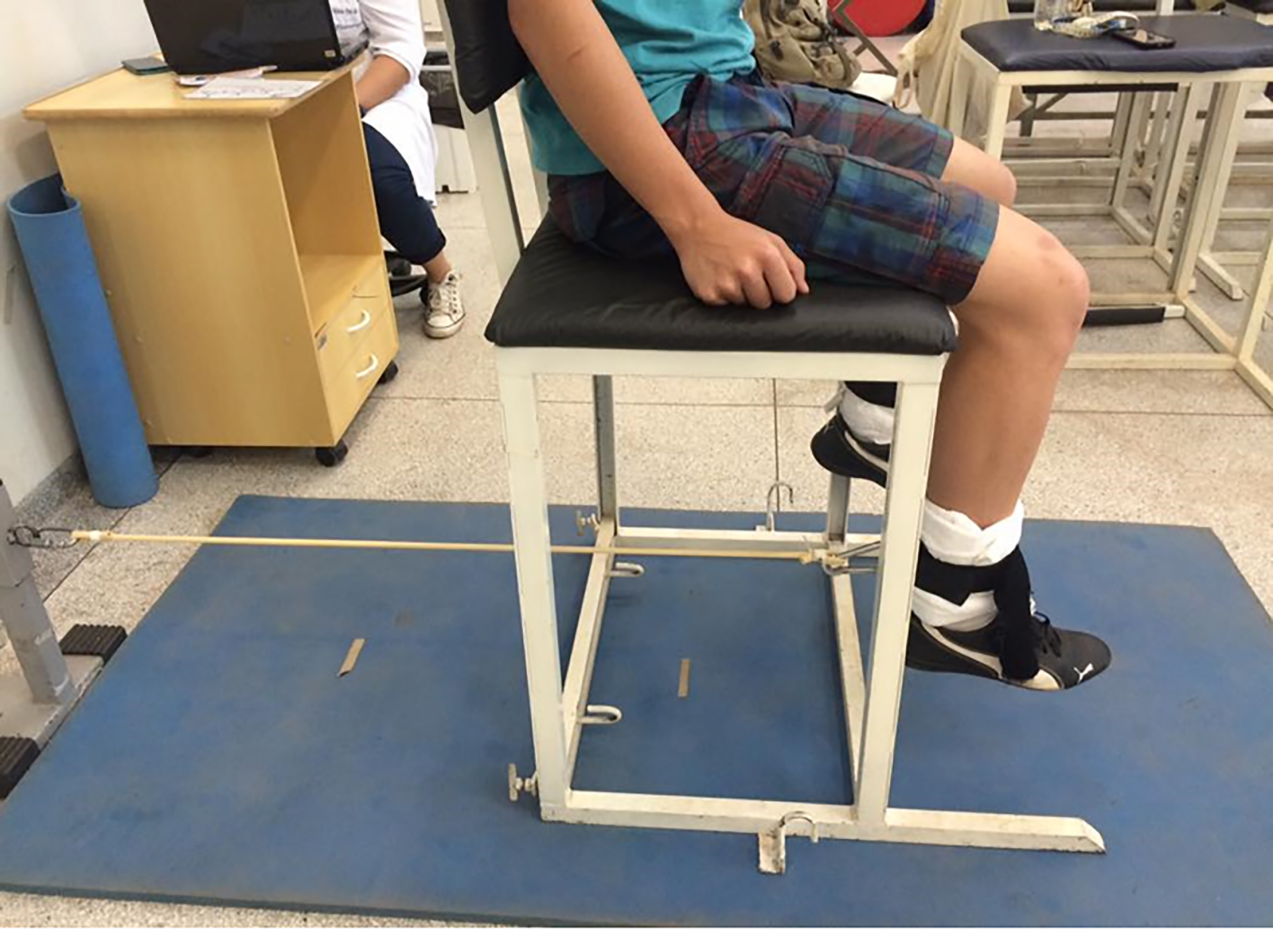
Positioning to perform the test.

All procedures were performed on a chair 69 cm tall. Adaptations were made regarding the height of the tube fixed on the bar, so the tube remained horizontal (Fig 3). The contralateral leg was immobilized on the chair with velcro. An elastic tube, 80 cm in length (Lemgruber—Rio de Janeiro—Brazil), was used for each participant, of which 10 cm was isolated at each end to build the handle. The tube thickness (11.5 x 6mm or 9x6mm) varied according to the individual tests.

The warm-up consisted of 20 seconds of flexion-extension of the dominant lower limb, with an easy load according to the OMNI-RES scale (2 points) [14]. In the first session of stage 3, the simulation of the test began with a fixed extension of 100 cm with the 9 x 6 mm tube. In the case of an invalid test (failure of stipulated time interval), two further attempts were allowed (S1 Chart). These extensions were tested with a 10–12 minute interval after application of the perceived recovery status scale (1–10). A maximum of two attempts was allowed in each session.

The procedures during the test and retest sessions were similar, starting with the warm-up followed by the test. A five-digit statistical digital counter (Western, São Paulo—Brazil) was used to assist the recording of repetitions, and a timer (Technos, Amazônia—Brazil) was used to monitor the time.

#### Perceived Exertion Scale for resistance exercise (OMNI-RES)

The perceived exertion was evaluated through the OMNI Perceived Exertion Scale for resistance exercise (OMNI-RES) [14], which consists of an ordinal scale ranging from 0 to 10, with 0 being extremely easy and 10 being extremely difficult. The following standardized question preceded the application of the scale: “How would you classify the intensity of the resistance in the exercise performed?”

#### Perceived recovery status scale

The perception of recovery of the dominant lower limb was assessed using the Perceived Recovery Status Scale of 1–10 points, one representing “no recovery” and 10 “fully recovered." The participant was asked the following question: "From 1 to 10 points, how do you classify the perception of recovery in the lower limb to take the test again now?" [17–19].

The scale was applied 10 minutes after the test. A new test began if the participant reported 8 points on the scale or reached a 12-minute interval between tests.

#### Selection of elastic tubes

All procedures were performed with elastic tubes (Lemgruber, Rio de Janeiro, Brazil) with an 11.5 x 6 mm or 9 x 6 mm caliber. The selection of elastic tubes took place before the test procedures. To guarantee homogeneity of the tubes and reliability of the results, all tubes were measured according to loads using a portable digital dynamometer (Instrutherm DD-300, São Paulo—Brazil).

Measurements were performed by a single participant who was positioned according to FRT standardizations. The participant was instructed to remain with their hands fixed laterally on the chair, trunk erect, and knee at 90° flexion (considered the point of exit). The participant was instructed to perform knee extension (0°) gradually at low speed. Three repetitions were performed with a 30-second interval between them. The peak value in kilograms (kg) was considered for the statistical analyses. A non-paired test was performed and elastic tubes with statistically significant differences (p<0.05) were excluded.

#### Statistical analysis

The statistical package SPSS (version 22; SPSS Inc, Chicago, IL) was used to conduct the analyses.

The descriptive profile of the sample is presented as mean and ​​standard deviation (SD) values. Data were analyzed after exclusion of values ​​greater than two SD according to time, as previously mentioned [20].

Relative reliability was calculated using the intraclass correlation coefficient (ICC), with 95% confidence interval (95%CI) from the mixed-effect model (2,1) [21]. The absolute reliability was verified with standard error of measurement (SEM) of each variable using the following formula: SD dif / √ 2 (where SD = standard deviation, dif = mean difference between tests). The absolute reliability was also expressed in coefficient of variation (CV), calculated using the following formula: (SEM / mean of tests) x 100 [22]. ICC was interpreted according to the following criteria: values ​​below 0.5 indicated low reliability, values ​​between 0.5 and 0.75 indicated moderate reliability, values between 0.75 and 0.9 indicated good reliability, and values ​greater than 0.90 indicated excellent reliability, as suggested by Koo et al. [21]. The paired t-test was used to verify differences within group between test and retest (p <0.05). All statistical analyzes assumed a significance level of 5%.

Homogeneity analysis regarding elastic tubes was expressed as mean and 95%CI.

## Results

Table 2 presents values of test, retest, and difference between test-retest [mean; SD; p-value; ICC (95%CI); SEM and CV (%)] of the participants according to time (seconds), repetitions (numerical sequence), and rhythm (repetitions/time). There were no significant differences in mean values between test and retest. Regarding ICC, there were differences for time (ICC = 0.66, 95%CI [0.50–0.76]), repetitions (ICC = 0.61, 95%CI [0.46–0.73]), and rhythm (ICC = 0.52, 95%CI [0.35–0.67]). The absolute reliability was analyzed by CV and showed the following values for time: CV(%) = 9.34; repetitions: CV(%) = 13.6; and rhythm: CV(%) = 10.2.

**Table 2:** Mean and SD of test-retest, p-value, intraclass coefficient of correlation, standard error of measurement, coefficient of variation.

	Test*	Retest*	*p-value*	ICC (95%CI)	SEM	CV (%)
Time(s)	54.32±8.69	54.82±8.71	0.527	0.66(0.50–0.76)	5.07	9.34
Repetitions	105.90±22.77	109.02±24.39	0.177	0.61(0.46–0.73)	14.59	13.6
Rhythm (s/repetition)	1.95±0.28	1.98±0.30	0.239	0.52(0.35–0.67)	0.20	10.2

*Values of mean and SD with 95% confidence interval. p-value>0.05; Legend: ICC: intraclass correlation coefficient; SEM: standard error of measurement; CV: coefficient of variation; SD: standard deviation; s: seconds.

There were no significant differences between the moments according to the OMNI-RES (Table 3).

**Table 3:** Mean and SD of perceived exertion scale (OMNI-RES) during all stages of the study.

	Learning 1	Learning 2	Test	Retest	*p-value*
*OMNI-RES*	7.4±1.2	7.0±1.1	7.3±0.9	7.3±0.8	0.133

Legend: N = 81 participants. SD: standard deviation. p-value > 0.05, with 95% confidence interval

## Discussion

The main findings of the study are related to the magnitude of errors between measurements. Thus, the relative reliability, verified through ICC, was classified as moderate. On the other hand, the absolute reliability, verified through SEM and expressed as CV, presented acceptable values.

Regarding the relative reliability, we observed lower ICC for rhythm when compared to the other variables. One possible explanation is the lack of speed control during the test due to the freedom to execute the movement. This finding is reinforced by Nyberg et al. [9], who suggested that different speeds can change reliability values in knee extensions. Moreover, this condition may have influenced the moment of muscular fatigue, with consequent reduction in the observed values. However, it is worth emphasizing that the model used, with limited systematization, was intended to reflect simple clinical conditions.

The absolute reliability was measured by SEM and also reported using CV(%), as suggested by other studies [22–26], in order to facilitate comparisons. Studies have described CV values > 10%, as reliable for this type of test, with no pre-defined time [25,27]. However, the value observed in the present study (9.34%) is below that previously mentioned, confirming the plausible values of clinical reliability.

Regarding time, studies have reported that the higher recurrent variability in protocols with fixed time to exhaustion is due to fatigue, boredom, or even lack of motivation during the performance [23, 26–29]. Laursen et al. [23] analyzed a race without predetermined time in athletes and adopted exhaustion as an interruption parameter. The reliability values of ICC and CV were 0.455 and 15.1, respectively, indicating higher variation in tests with freedom of execution until fatigue. We highlight that the control measurements adopted in our study seem to have attenuated such effects, ensuring better results.

The scientific literature does not currently present results regarding tests of muscular resistance using elastic bands. This could be due to the possibility of large variations in time and free rhythm of execution, with consequent reduction in reliability when compared to options that adopt predetermined time. However, it is necessary to consider whether the concept of fatigue resistance would apply in tests with a predetermined time, as the potential of the individual being tested would be underestimated.

Findings concerning repetition showed values of ICC = 0.66 and CV = 13.6%. A possible explanation is the absence of linear rhythm during the test, as previously mentioned. It is believed that such a mechanism may have influenced the regularity and control of the repetitions performed.

Regarding the strategy of prescription used, the lack of intentional control of speed during the test showed negative repercussions for the analyzed variables. However, despite this, the results presented acceptable values, since the main limitation, referring to control of speed was intentional, as mentioned above.

Some aspects in the manuscript collaborate and guarantee acceptable reliability with regard to the proposed test. First, unlike the other studies cited [8–13], the FRT respects the principle of the participants' biological individuality. Second, the protocol design used was elaborated with strict methodological quality and we inserted, for example, 2 familiarization sessions in order to minimize possible bias. Third, the statistical analysis used was based on the ideal of quality and reference studies in the area [22,28,30]. Therefore, the reasons given demonstrate the use of a well-defined theoretical basis, a fact that reiterates the quality and veracity of the presented results.

The OMNI-RES scale were performed to control perceived exertion during the stages of the study, and the absence of statistical differences between the moments of intervention suggests, although subjectively, a tendency of similarity throughout the study. Perceived exertion and recovery deserves attention when considering the increase in loads. Regarding exercise prescription, the use of elastic tools must respect safe and acceptable loads, considering the elastic properties and individual capacity. However, studies [14,31] emphasize the difficulty of controlling this type of training, highlighting the need for robust and well-applied methodologies.

When considering the current evidence on this issue, the difficulty in quantifying elastic resistance usually reflects an important limitation of controlling the intensity in periodized rehabilitation programs. Thus, the strategy suggests the use of a model in which, even with variation in loads between individuals, the volume of work is coherent with the capacity of each subject to generate strength, resulting in an individualized stress zone and better levels of adaptation. The importance of the proposed strategy for rehabilitation programs is highlighted due to the use of the quadriceps muscles, which present valuable function related to the execution of daily activities [32].

As far as we know, this is the first study to verify the reliability of a resistance test using elastic tubes. Previously, six studies have demonstrated the reliability of strength tests using elastic resistance, which showed higher reliability values [8–13]​​. However, the dynamics, methodological procedures, and variables of those studies were different, which may justify the differences in results.

The strengths of the study include the high number of participants included and the simple reproduction of the FRT. Additionally, it is worth mentioning that the elastic tubes used in the first stages were replaced by new ones to avoid possible influences of mechanical changes over time. We suggest the development of studies that explore the physiological analysis of FRT, distinct muscle groups, and different population profiles in order to elaborate methods that are reliable, accessible, and inexpensive.

## Conclusions

In summary, the muscle fatigue resistance test using elastic tubes demonstrated evidence of moderate reliability. The perspectives regarding the findings of the present study suggest the application of the test in diverse scenarios, using few resources and accessible logistics, which include the sports area.

## Supporting information

S1 Chart Possible extensions. (TIF)

## References

[1] Martins WR, Oliveira RJ, Carvalho RS, Damasceno VO, Silva VZ, Silva MS. Elastic resistance training to increase muscle strength in elderly: a systematic review with meta-analysis. Arch Gerontol Geriatr. 2013; 57(1):8–15. 10.1016/j.archger.2013.03.002 23562413

[2] Jensen J, Hölmich P, Bandholm T, Zebis MK, Andersen LL, Thorborg K. Eccentric strengthening effect of hip-adductor training with elastic bands in soccer players: a randomised controlled trial. Br J Sports Med.2014; 48(4):332–8. 10.1136/bjsports-2012-091095 22763117

[3] Jakobsen MD, Sundstrup E, Andersen CH, Persson R, Zebis MK, Andersen LL. Effectiveness of hamstring knee rehabilitation exercise performed in training machine vs. elastic resistance: electromyography evaluation study. Am J Phys Med Rehabil. 2014; (93): 320–327. 24398577 10.1097/PHM.0000000000000043

[4] Aboodarda SJ, Page PA, Behm DG. Muscle activation comparisons between elastic and isoinertial resistance: A meta-analysis. Clin Biomech. 2016; 39: 52–61. 10.1016/j.clinbiomech.2016.09.008 27681867

[5] Colado JC, Triplett T. Effects of a short-term resistance program using elastic bands versus weight machines for sedentary middle-aged women. J Strength Cond Res. 2008; 1441–1448. 10.1519/JSC.0b013e31817ae67a 18714245

[6] Sundstrup E, Jakobsen MD, Andersen CH, Jay K, Persson R, Aagaard P, et al Participatory ergonomic intervention versus strength training on chronic pain and work disability in slaughterhouse workers: study protocol for a single-blind, randomized controlled trial. BMC Musculoskelet Disord 2013; 14:67 10.1186/1471-2474-14-67 23433448 PMC3606231

[7] Metgud S, Dalal P, Joshi P. Effect of soccer trainer and elastic band on quadríceps femoris muscle strengt in Young healthy individuals: a randomized controlled trial. Int J Physiother Res. 2015; 3(3):1091–97. doi: 10.16965/ijpr.2015.118

[8] Guex K, Daucourt C, Borloz S. Validity and reliability of maximal-strength assessment of knee flexors and extensors using elastic bands. J Sport Rehabil. 2015; 24(2):151–155. 10.1123/jsr.2013-0131 24700494

[9] Nyberg A, Lindström B, Aronsson N, Näslund M, Wadell K. Validity of using Elastic Bands to Measure Knee Extension Strength in Older Adults. J Nov Phys Rehabil. 2016; 3(1): 016–021. doi: 10.17352/2455-5487.000030

[10] Newsam CJ, Leese C, Fernandez-Silva J. Intratester reliability for determining an 8-repetition maximum for 3 shoulder exercises using elastic bands. J Sport Rehabil. 2005; 14:35–47. 10.1123/jsr.14.1.35.

[11] Augustsson J. A new clinical muscle function test for assessment of hip external rotation strength: augustosson stregth test. Int J Sports Phys Ther. 2016; 11(4): 520 27525176 PMC4970842

[12] Manor B, Topp R, Page P. Validity and Reliability of Measurements of Elbow Flexion Strength Obtained from Older Adults Using Elastic Bands. J Geriatr Phys Ther. 2006; 29(1):18–21. 16630372

[13] Andersen LL, Vinstrup J, Jakobsen MD, Sundstrup E. Validity and reliability of elastic resistance bands for measuring shoulder muscle strength. Scand J Med Sci Sports. 2016;27(8):887–894. 10.1111/sms.12695 27185407

[14] Colado JC, Garcia-Masso X, Triplett NT, Flandez J, Borreani S, Tella. Concurrent validation of the OMNIResistance exercise scale of perceived exertion with Thera-Band resistance bands. J Strength Cond Res. 2012; 26:3018–3024. 10.1519/JSC.0b013e318245c0c9 22210471

[15] Wells GD, Selvadurai H, Tein I. Bioenergetic provision of energy for muscular activity. Paediatr Respir Ver. 2009;10(3):83–90. 10.1016/j.prrv.2009.04.005 19651377

[16] Ramos EMC, Toledo-Arruda AC, Fosco LC, Bonfim R, Bertolini GN, Guarnier FA, et al The effects of elastic tubing-based resistance training compared with conventional resistance training in patients with moderate chronic obstructive pulmonary disease: a randomized clinical trial. Clin Rehabil. 2014; 28(11): 1096–1106. 10.1177/0269215514527842 24647863

[17] Almeida AC, Machado AF, Albuquerque MC, Netto LM, Vanderlei FM, Vanderlei LCM, et al The effects of cold water immersion with different dosages (duration and temperature variations) on heart rate variability post-exercise recovery: A randomized controlled trial. Med Sci Sports Exerc. 2016; 19: 676–68. 10.1016/j.jsams.2015.10.003 26614422

[18] Machado AF, Almeida AC, Micheletti JK, Vanderlei FM, Tribst MF, Netto Junior J, et al Dosages of cold water immersion post-exercise on functional and clinical responses: a randomized controlled trial. Scand J Med Sci Sports. 2016; [ahead of print]. 10.1111/sms.12734 27430594

[19] Buchheit M, Peiffer JJ, Abbiss CR, Laursen PB. Effect of cold water immersion on postexercise parasympathetic reactivation. Am J Physiol Heart Circ Physiol. 2009; 296:421–427. 10.1152/ajpheart.01017.2008 19074671

[20] Bland JM, Altman DG. Statistical methods for assessing agreement between two methods of clinical measurement. Lancet. 1986; 1(8476): 307–310. 2868172

[21] Koo TK, Mae YL. A Guideline of Selecting and Reporting Intraclass Correlation Coefficients for Reliability Research. J Chiropr Med. 2016; 15:155–163. 10.1016/j.jcm.2016.02.012 27330520 PMC4913118

[22] Hopkins WG. Measures of Reliability in Sports Medicine and Science. Sports Med 2000; 30 (1): 1–15. 10907753 10.2165/00007256-200030010-00001

[23] Laursen PBGT, Francis CR, Abbiss MJ, Newton K. Reliability of Time-to-Exhaustion versus Time-Trial Running Tests in Runners. Med Sci Sports Exerc. 2007; 39 (8):1374–1379. 10.1249/mss.0b013e31806010f5 17762371

[24] Ferri-Morales S, Alegre LM, Basco A, Aguado X. Test-retest relative and absolute reliability of knee extensor strength measures and minimal detectable change. Isokinet Exerc Sci. 2014; 22:17–26.

[25] Pageaux B, Lepers R, Marcora SM. Reliability of a Novel High Intensity One Leg Dynamic Exercise Protocol to Measure Muscle Endurance. Plos One. 2016; 11(10): e0163979 10.1371/journal.pone.0163979 27706196 PMC5051904

[26] Lubans DR, Morgan P, Callister R, Plotnikoff RC, Eather N, Riley N, et al Test–retest reliability of a battery of field-based health-related fitness measures for adolescentes. J Sport Health Sci. 2011; 29(7): 685–693. 10.1080/02640414.2010.551215 21391082

[27] Jeukendrup AE, Currell K. Should time trial performance be predicted from three serial time-to-exhaustion tests? Med Sci Sports Exerc. 2005; 37(10):1821. 10.1249/01.mss.0000175095.56646.4b 16260987

[28] Hopkins WG, Schabort EJ, Hambley JA. Reliability of power in physical performance tests. Sports Med. 2001; 31:211–234. 11286357 10.2165/00007256-200131030-00005

[29] Currell K, Jeukendrup AE. Validity, reliability and sensitivity of measures of sporting performance. Sports Med. 2008; 38(4):297–316. 10.2165/00007256-200838040-00003 18348590

[30] Atkinson G, Nevill AM. Statistical methods for assessing measurement error (reliability) in variables relevant to sports medicine. Sports Med. 1998; 26(4):217–38. 9820922 10.2165/00007256-199826040-00002

[31] Ghigiarelli JJ, Nagle EF, Gross FL, Robertson RJ, Irrgang JJ, Myslinski T. The effects of a 7-week heavy elastic band and weight chain program on upper-body strength and upper-body power in a sample of division 1-AA football players. J Strength Cond Res. 2009; 23(3): 756–764. 10.1519/JSC.0b013e3181a2b8a2 19387404

[32] Pappas E, Nightingale EJ, Simic M, Ford KR, Hewett TE, Myer GD. Do exercises used in injury prevention programmes modify cutting task biomechanics? A systematic review with meta-analysis. Br J Sports Med. 2015: 49(10):673–80. 10.1136/bjsports-2014-093796 25492646

