# Supplementary figures and images for: Test-retest reliability of knee extensors endurance test with elastic resistance
Source: PLoS One. 2018 Aug 31;13(8):e0203259. doi: 10.1371/journal.pone.0203259 (PMC6118382; doi:10.1371/journal.pone.0203259)

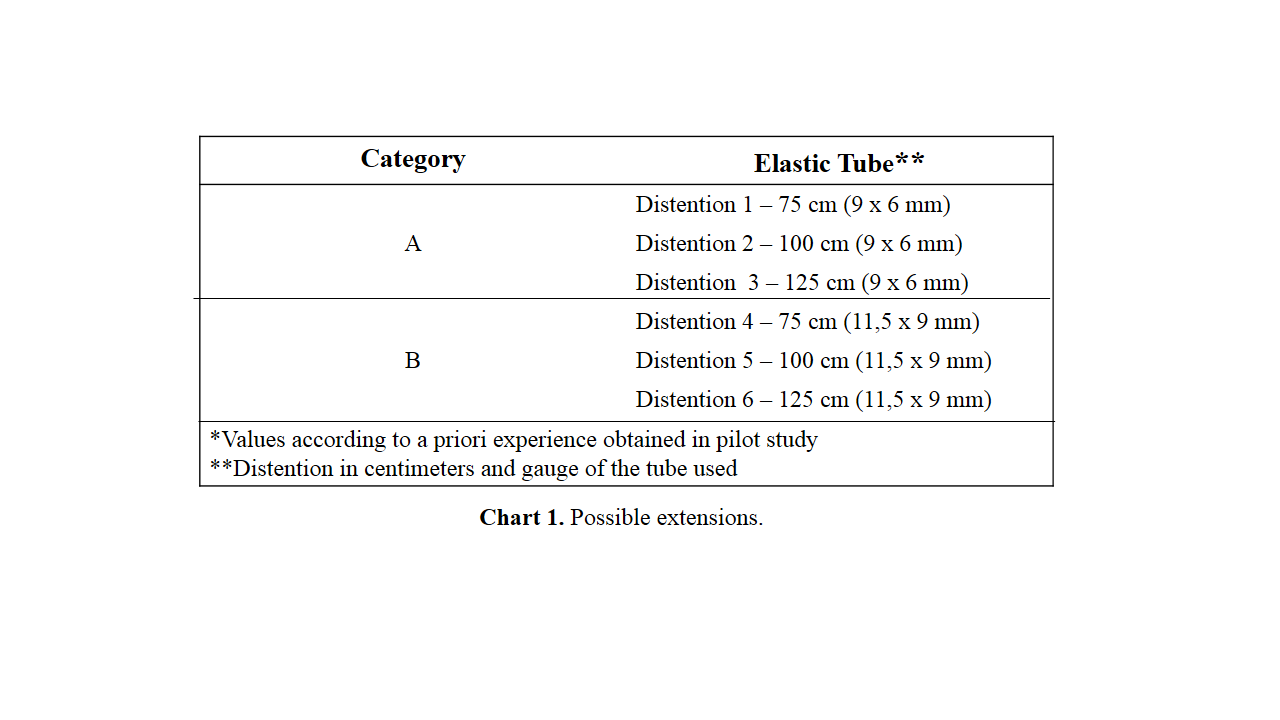

Supplement: S1 Chart — (TIF) [file pone.0203259.s001.tif]
